# Estrogen therapy induces an unfolded protein response to drive cell death in ER+ breast cancer

**DOI:** 10.1002/1878-0261.12528

**Published:** 2019-07-09

**Authors:** Sarah R. Hosford, Kevin Shee, Jason D. Wells, Nicole A. Traphagen, Jennifer L. Fields, Riley A. Hampsch, Arminja N. Kettenbach, Eugene Demidenko, Todd W. Miller

**Affiliations:** ^1^ Department of Molecular & Systems Biology Norris Cotton Cancer Center Geisel School of Medicine at Dartmouth Lebanon NH USA; ^2^ Department of Microbiology and Immunology Norris Cotton Cancer Center Geisel School of Medicine at Dartmouth Lebanon NH USA; ^3^ Department of Biochemistry Norris Cotton Cancer Center Geisel School of Medicine at Dartmouth Lebanon NH USA; ^4^ Department of Biomedical Data Sciences Norris Cotton Cancer Center Geisel School of Medicine at Dartmouth Lebanon NH USA; ^5^ Comprehensive Breast Program Norris Cotton Cancer Center Geisel School of Medicine at Dartmouth Lebanon NH USA

**Keywords:** anti‐estrogen, breast cancer, endocrine, estrogen receptor, resistance

## Abstract

Estrogens have been shown to elicit anticancer effects against estrogen receptor α (ER)‐positive breast cancer. We sought to determine the mechanism underlying the therapeutic response. Response to 17β‐estradiol was assessed in ER+ breast cancer models with resistance to estrogen deprivation: WHIM16 patient‐derived xenografts, C7‐2‐HI and C4‐HI murine mammary adenocarcinomas, and long‐term estrogen‐deprived MCF‐7 cells. As another means to reactivate ER, the anti‐estrogen fulvestrant was withdrawn from fulvestrant‐resistant MCF‐7 cells. Transcriptional, growth, apoptosis, and molecular alterations in response to ER reactivation were measured. 17β‐estradiol treatment and fulvestrant withdrawal induced transcriptional activation of ER, and cells adapted to estrogen deprivation or fulvestrant were hypersensitive to 17β‐estradiol. ER transcriptional response was followed by an unfolded protein response and apoptosis. Such apoptosis was dependent upon the unfolded protein response, p53, and JNK signaling. Anticancer effects were most pronounced in models exhibiting genomic amplification of the gene encoding ER (*ESR1*), suggesting that engagement of ER at high levels is cytotoxic. These data indicate that long‐term adaptation to estrogen deprivation or ER inhibition alters sensitivity to ER reactivation. In such adapted cells, 17β‐estradiol treatment and anti‐estrogen withdrawal hyperactivate ER, which drives an unfolded protein response and subsequent growth inhibition and apoptosis. 17β‐estradiol treatment should be considered as a therapeutic option for anti‐estrogen‐resistant disease, particularly in patients with tumors harboring *ESR1* amplification or ER overexpression. Furthermore, therapeutic strategies that enhance an unfolded protein response may increase the therapeutic effects of ER reactivation.

**Abbreviations**
AI: aromatase inhibitor
EDC: estrogen–dendrimer conjugate
EnR: endoplasmic reticulum
ERE: estrogen response element
ER: estrogen receptor alpha
*ESR1*: gene encoding estrogen receptor alpha
FR: fulvestrant‐resistant
Fulv: fulvestrant
GSVA: gene set variation analysis
HER2: human epidermal growth factor receptor 2 (*ERBB2*)
IHC: immunohistochemistry
LTED: long‐term estrogen‐deprived
SRA: Sequence Read Archive

## 1 Introduction

Breast cancer is the most commonly diagnosed female cancer and the second‐leading cause of cancer‐related death in women in the United States. Due to the predominance of the estrogen receptor α (ER)‐positive/HER2‐negative (ER+/HER2−) subtype (~ 60% of cases), these tumors account for more recurrences and deaths than all other subtypes combined. Adjuvant anti‐estrogen therapies that antagonize ER directly (e.g., tamoxifen) or block estrogen biosynthesis [e.g., aromatase inhibitors (AIs)] have improved outcomes in patients with ER+ disease (Sledge *et al*., 2014). However, resistance to anti‐estrogens is common: Within 15 years of initial diagnosis, approximately 33% of patients treated with adjuvant anti‐estrogen therapy present with recurrent disease (Early Breast Cancer Trialists’ Collaborative Group *et al*., 2011; Ferlay *et al*., 2010).

Prior to the approval of the pioneer anti‐estrogen tamoxifen, estrogens (e.g., diethylstilbestrol) were used to treat patients with breast cancer. In the advanced/metastatic setting of treatment‐naïve breast cancer, estrogens provided response rates similar to those obtained with tamoxifen, and diethylstilbestrol provided longer overall survival than tamoxifen (Ingle *et al*., 1981; Peethambaram *et al*., 1999; Stewart *et al*., 1980). Clinical analyses showed that longer duration of the postmenopausal period as well as longer‐term treatment with anti‐estrogen therapy was associated with enhanced therapeutic effects of estrogens on breast tumors (Ellis *et al*., 2009; Haddow *et al*., 1944; Lonning *et al*., 2001; Zucchini *et al*., 2015). Similarly, withdrawal/cessation of anti‐estrogen therapy has shown antitumor effects, suggesting that reactivation of ER may be therapeutic (Agrawal *et al*., 2011; Canney *et al*., 1987; Howell *et al*., 1992).

Preclinical work has shown that anti‐estrogen‐resistant ER+ breast cancer cell lines and xenografts can become sensitized to anti‐estrogen withdrawal and estrogen treatment (reviewed by Jordan *et al*., 2011). However, preclinical studies to characterize the mechanism(s) underlying therapeutic response to estrogens have thus far been limited to derivatives of two ER+ breast cancer cell lines. Evidence supports mechanisms that include ER‐driven upregulation of genes involved in apoptosis, inflammation, and endoplasmic reticulum (EnR) stress (Ariazi *et al*., 2011; Fan *et al*., 2018; Lewis *et al*., 2005; Obiorah *et al*., 2014; Song *et al*., 2001). We evaluated multiple additional *in vivo* models of therapeutic response to estrogen and further deciphered a cellular mechanism underlying response to ER reactivation therapy. Mechanistic understanding of how estrogens are growth stimulatory in one context and growth inhibitory in another will facilitate the development of biomarkers predictive of therapeutic response and reveal targetable pathways to enhance the anticancer effects of ER reactivation.

## 2 Materials and methods

### 2.1 Cell culture

MCF‐7 cells were obtained from American Type Culture Collection (ATCC; Manassas, VA, USA) and cultured in DMEM/10% FBS (HyClone, GE Healthcare Bio‐Sciences, Pittsburgh, PA, USA). Fulvestrant‐resistant MCF‐7 (FR) cells and long‐term estrogen‐deprived MCF‐7 (LTED) cells were gifts from Matthew Ellis (Baylor College of Medicine, Houston, TX, USA; Sanchez *et al*., 2011). FR cells were maintained in DMEM/10% FBS containing 1 μm fulvestrant (fulv; Tocris, Bio‐Techne Corp., Minneapolis, MN, USA). LTED cells were maintained in phenol‐red‐free DMEM with 10% dextran‐coated charcoal‐treated FBS (DCC‐FBS; HyClone) and were passaged using phenol‐red‐free 0.25% trypsin plus 2.21 mm EDTA (Corning, Tewksbury, MA, USA).

Cell lines were authenticated by STR genotyping at the University of Vermont Cancer Center DNA Analysis Facility. Cell lines were confirmed to be negative for mycoplasma and passaged for < 4 months before experimentation. Cells were treated ± 17β‐estradiol (E2; Sigma, St. Louis, MO, USA), estrogen–dendrimer conjugate (EDC) (prepared as described in ref. Harrington *et al*., 2006), empty dendrimer, KIRA6 (Cayman Chemical, Ann Arbor, MI, USA), or PD‐0332991 (palbociclib; Selleck Chemicals, Houston, TX, USA).

### 2.2 Immunoblotting

Immunoblotting of protein extracts from cells and frozen tumor tissue was performed as previously described (Shee *et al*., 2018). Cells were lysed, and tumors were homogenized in cold RIPA buffer (50 mm Tris pH 7.4, 150 mm NaCl, 1% NP‐40, 0.5% deoxycholic acid, 0.1% SDS, 1 mm EDTA, 1 mm EGTA, 5 mm sodium pyrophosphate, 50 mm NaF, 10 mm β‐glycerophosphate) containing protease inhibitors (Halt Protease Inhibitor Cocktail; Pierce, Thermo Fisher, Waltham, MA, USA) and phosphatase inhibitor (1 mm Na_3_VO_4_; New England Biolabs, Ipswich, MA, USA). Lysates were sonicated for 15 s and centrifuged at 14 000 ***g*** for 10 min at 4 °C, and supernatant protein concentration was quantified by BCA assay (Pierce). Protein extracts were denatured and reduced with NuPAGE (Life Technologies, Carlsbad, CA, USA) containing 1.25% β‐mercaptoethanol (Sigma). SDS/PAGE‐separated proteins were transferred to nitrocellulose, and Ponceau S stain was used to visually confirm even protein loading/transfer. Blots were probed with primary antibodies against actin, vinculin, PARP, IRS‐1, IGF‐1Rβ, NFκB p65, IκBα, P‐4EPB1_T37/46_, P‐S6_S240/244_, P‐p70S6K_T389_, IRE1α, PERK, SAPK/JNK, P‐SAPK/JNK_T183/Y185_, Ero1‐Lα, PDI, Bip, P‐cJUN_S73_, Bim, CHOP, lamin A, P‐ATF2_T71_, P‐SEK/MKK4_S257_ (Cell Signaling, Danvers, MA, USA), p53, and ERα (Santa Cruz Biotechnology, Dallas, TX, USA). Signal was detected with HRP‐labeled secondary mouse or rabbit antibodies (GE Healthcare, Pittsburg, PA, USA) and ECL substrate (Pierce). Experiments were performed at least twice; representative results are shown.

### 2.3 Nuclear/cytoplasmic protein extraction

Cells were pretreated as indicated and then reseeded in 60‐mm dishes at 10^6^ cells/dish. The next day, cells were treated ± 1 nm E2 for 4 or 24 h. Cells were then harvested using trypsin‐EDTA and centrifuged at 500 ***g*** for 5 min. Cell pellets were washed with PBS; then, cytoplasmic and nuclear proteins were extracted using the NE‐PER kit (Thermo Fisher). Following isolation of nuclear and cytoplasmic fractions, protein concentrations were measured by BCA assay (Pierce), and proteins were denatured, reduced, and analyzed by immunoblot.

### 2.4 Apoptosis assay

All treatment conditions were assayed in triplicate in 12‐well plates: 2 × 10^4^ cells/well were seeded, treated as indicated for 4 days, and then assayed. Apoptosis of adherent and nonadherent cells was measured by flow cytometry using the ApoScreen Annexin V and Propidium Iodide Kit (Southern Biotech, Birmingham, AL, USA) as per manufacturer's instructions.

### 2.5 Colony formation assay

Cells were seeded in triplicate in 6‐well (10^4^/well), 12‐well (0.5 × 10^4^/well), or 96‐well (10^3^/well) plates and then treated as indicated for up to 4 weeks. When the most confluent well reached ~ 90% confluence, cells were fixed and stained with 20% methanol/80% water/0.5% crystal violet for 10 min and washed with water. Dried plates were scanned, and stain intensity was quantified using the ColonyArea plugin in imagej (Guzman *et al*., 2014).

### 2.6 Short‐term growth assay

Cells were pretreated as indicated and then reseeded at 10^3^ cells/well in triplicate in 96‐well plates. Cells were treated as indicated, and after 5 days, cells were fixed and relative viable cell numbers were determined by sulforhodamine B (SRB) staining as described (Vichai and Kirtikara, 2006).

### 2.7 Senescence‐associated β‐galactosidase assay

MCF‐7 and FR cells were treated as indicated and then reseeded in 6‐well plates in triplicate at 1 week prior to assay. Senescence‐associated β‐galactosidase staining was performed as per manufacturer's instructions (Cell Signaling). Proportions of positively stained cells were counted in three microscopic fields (400× magnification).

### 2.8 Luciferase transcriptional reporter assay

Plasmids encoding firefly luciferase under the control of promoters driven by an estrogen response element (ERE; a gift from Dorraya El‐Ashry, University of Minnesota), an NFκB element (Addgene, Watertown, MA, USA, catalog #49343), a p53 element (Addgene catalog #16442), or a cJUN element (Addgene catalog #40342) were cotransfected into cells along with a plasmid encoding CMV‐Renilla luciferase (Promega, Madison, WI, USA, catalog #E2261) as a transfection control. The next day, cells were treated as indicated, and luciferase activities were subsequently measured using the Dual‐Luciferase Reporter Assay System (Promega) as per manufacturer's instructions. Firefly values were normalized to Renilla controls.

### 2.9 RNA interference

Cells were transfected using Lipofectamine RNAiMAX (Life Technologies) and 20 μm siRNA targeting *ESR1* (ERα, Qiagen, Hilden, Germany, catalog #SI02781401), *ERN1* (IRE1α; Dharmacon, GE Healthcare Bio‐Sciences, catalog #L‐004951‐02‐0005), *EIF2AK3* (PERK; Dharmacon catalog #L‐004883‐00‐0005), *TP53* (p53; Dharmacon catalog #L‐003329‐00‐0005), or *MAPK8* (JNK, Dharmacon catalog #L‐003514‐00‐0005). Cells were then reseeded into 12‐well plates (2 × 10^4^ cells/well for apoptosis assays) and 6‐well plates (0.5–1 × 10^6^ cells/wells for immunoblotting). The next day, media was refreshed. At 2 days post‐transfection, cells were treated as indicated. After 4 days of treatment, cells were harvested for apoptosis assay, or protein was harvested for immunoblot.

### 2.10 Mouse studies

Studies were approved by the Dartmouth College IACUC. Female NOD.Cg‐Prkdc^scid^Il2rg^tm1Wjl^/SzJ (NSG) mice (4–5 weeks old; obtained from the Norris Cotton Cancer Center Mouse Modeling Shared Resource) were ovariectomized and implanted subcutaneously (s.c.) with ~ 8‐mm^3^ fragments of serially transplanted WHIM16 patient‐derived xenograft (PDX) breast tumor tissue [obtained from the Washington University HAMLET Core (Puenpa *et al*., 2013)]. Female BALB/cJ mice (4–5 weeks old; obtained from Jackson Laboratory) were ovariectomized and implanted s.c. with fragments of serially transplanted C4‐HI or C7‐2‐HI murine mammary adenocarcinoma tissue [gifts from Claudia Lanari, Consejo Nacional de Investigaciones Científicas y Técnicas, Buenos Aires, Argentina (Kordon *et al*., 1991; Soldati *et al*., 2010; Vanzulli *et al*., 2005)]. In all mice, tumor volume was measured twice weekly using calipers (volume = width^2^ × length/2). When tumors reached ~ 400 mm^3^, mice were randomized to receive sham surgery or s.c. implantation with an E2 pellet (0.72 mg, 60‐day release; Innovative Research of America, Sarasota, FL, USA). For molecular analyses, tumors were harvested at the indicated time points and cut into pieces for snap‐freezing.

### 2.11 SNP microarrays

Genomic DNA was extracted from MCF‐7 and FR cells using the DNeasy Blood and Tissue Kit (Qiagen). DNA was labeled and hybridized to Affymetrix (Santa Clara, CA, USA) SNP 6.0 arrays, and arrays were scanned at Coriell Institute for Medical Research (Camden, NJ, USA) as per manufacturer's instructions. genotyping console software (Affymetrix) was used to analyze data, generate copy number results (CNCHP files), and determine log_2_ copy number of each genomic region evaluated. Data are deposited at NCBI Gene Expression Omnibus (GEO) under accession # GSE121631.

### 2.12 Gene expression microarrays

Fulvestrant‐resistant cells maintained in DMEM/10% FBS + 1 μm fulv were treated with fulv withdrawal (FW) for 0–14 days in triplicate in 100‐mm dishes. RNA was extracted in 2‐day intervals using RiboZol (VWR, Radnor, PA, USA). RNA was used for expression profiling with Illumina (San Diego, CA, USA) HumanHT‐12 v4 Expression BeadChips as per manufacturer's instructions. Data were processed by stabilizing transformation and robust spline normalization using the lumi package in r software (https://www.r-project.org/). Data are deposited at NCBI GEO under accession # GSE121379.

### 2.13 RNA sequencing

MCF‐7 and LTED cells were treated with DMEM/10% DCC‐FBS for 3 days and then treated ± 1 nm E2 × 7 days in triplicate in 100‐mm dishes. Mice bearing WHIM16 or C7‐2‐HI tumors were treated ± E2 via s.c. pellet for 3 days, followed by harvest and freezing of tumor fragments. RNA was extracted using RNeasy Plus Mini Kit (Qiagen Cat 1062832) and QIAzol Lysis Reagent (Qiagen Cat 1023537). RNA quality was assessed on a fragment analyzer (Advanced Analytical Technologies, Agilent, Santa Clara, CA, USA), and RNA was quantified by Qubit. In preparation for RNA sequencing (RNA‐seq), ribo‐depleted libraries were prepared from 2.5 μg of total RNA using the Globin‐Zero Gold (catalog # GZG1206; Illumina) and TruSeq Stranded Total RNA (catalog # RS‐122‐2201; Illumina) workflows according to manufacturer's instructions. Each library was uniquely barcoded, quantified by qPCR (catalog # KK4824; Kapa Biosystems, Wilmington, MA, USA), and pooled for sequencing on an Illumina NextSeq 500 (2 × 75‐bp). Reads were checked for quality control using fastqc (Andrews, 2010) and if necessary were trimmed using Trimmomatic (Bolger *et al*., 2014) to trim regions with phred Q > 30 (Ewing *et al*., 1998). High‐quality reads were then aligned to reference genome hg19 using star (Dobin *et al*., 2013). Gene counts were normalized by frequency per kilobase million (Garber *et al*., 2011). Differential expression of genes was determined using the limma (Ritchie *et al*., 2015) and DESeq2 (Love *et al*., 2014) packages in the R environment (R Core Team, 2017), and multiple testing correction was performed using the FDR Benjamini–Hochberg method (Benjamini and Hochberg, 1995). Genes were determined to be significantly differentially expressed if FDR *q *≤* *0.05 and absolute log_2_ fold change ≥ 1. To determine significant gene expression pathway enrichment between time points, unsupervised sample‐wise enrichment analysis of hallmark pathways using gene set variation analysis (GSVA) (Hanzelmann *et al*., 2013) was performed in R using default arguments with an adjusted *P*‐value significance threshold of 0.25 (Subramanian *et al*., 2005). RNA‐seq data were deposited at NCBI Sequence Read Archive (SRA) under accession #: PRJNA497539.

### 2.14 Proteasomal activity assay

Cells were plated in triplicate in 96‐well plates at 5 × 10^3^ cells/well and then treated as indicated. Live‐cell proteasomal activity was assayed using the Proteasome‐Glo Chymotrypsin‐like Cell‐based Assay (Promega) as per manufacturer's instructions. Luciferase activity was normalized to relative cell number as measured using an S3 Live Cell Analysis System (IncuCyte, Essen BioScience, Ann Arbor, MI, USA).

### 2.15 qPCR and RT‐qPCR

Cells were seeded in 60‐mm dishes and treated as indicated in triplicate. For RT‐qPCR analysis, RNA was extracted using the RNeasy Plus Mini Kit (Qiagen) and treated with DNase I; then, cDNA was synthesized using the iScript cDNA Synthesis Kit (Bio‐Rad, Hercules, CA, USA). For genomic DNA analysis, DNA was extracted using the DNeasy Blood and Tissue Kit (Qiagen). Real‐time qPCR was performed using iQ SYBR Green Supermix (Bio‐Rad) with the following primers:

RT‐qPCR primers:

**Table:** 

Target gene	Forward primer	Reverse primer
CDKN1A (p21)	TGAGCCGCGACTGTGATG	GTCTCGGTGACAAAGTCGAAGTT
BBC3 (PUMA)	ACCTCAACGCACAGTACGAG	CCCATGATGAGATTGTACAGGA
PMAIP1 (NOXA)	AAGAAGGCGCGCAAGAAC	TCCTGAGCAGAAGAGTTTGGT
36B4	GTGTTCGACAATGGCAGCAT	GACACCCTCCAGGAAGCGA

Genomic DNA primers:

**Table:** 

Target gene	Forward primer	Reverse primer
Human *ESR1*	CCATGACCCTCCACACC	CTCGTTCCCTTGGATCTGA
Human *ASXL1*	CAGCTTCTCACTTGGCCTTC	GCTCTGCACAGGACAGATCA
Mouse *Esr1*	TTGAACTTGTCCCCTTGACC	ACAGGTGGCGCTCTGAAA
Mouse *Asxl1*	AGATCACACTACCTCCAAAGTGC	TCCAAAGGAGAGGCTCACA

## 3 Results

### 3.1 Restoration of ER signaling in anti‐estrogen‐resistant breast tumors and cancer cells elicits therapeutic effects

**Figure 1:**
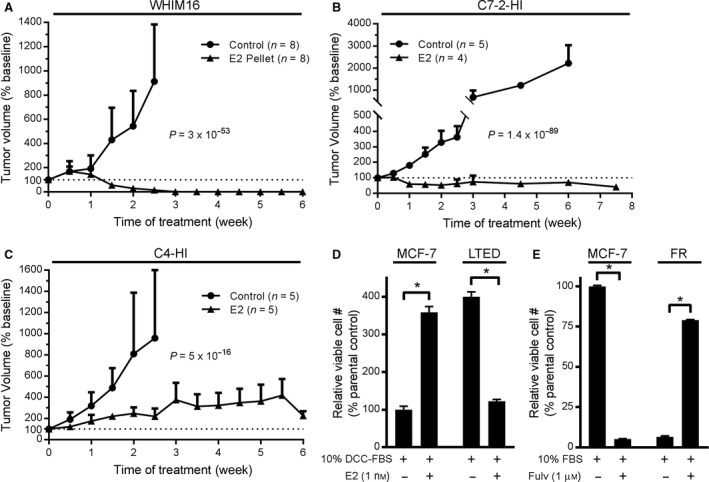
Estrogen‐independent tumors and cells exhibit therapeutic sensitivity to ER reactivation via E2 treatment or anti‐estrogen withdrawal. (A–C) Ovariectomized NSG (A) or BALB/cJ (B, C) mice were implanted s.c. with ~ 8‐mm^3^ fragments of serially transplanted WHIM16 patient‐derived xenografts (A), C7‐2‐HI murine allografts (B), or C4‐HI murine allografts (C). When tumors reached ~ 400 mm^3^, mice were randomized to sham surgery or s.c. implantation with an E2 pellet. Tumor volume is presented as mean + SD of % change from baseline on the day of E2 supplementation (Week 0). Groups were compared by linear mixed modeling. (D) MCF‐7 and LTED cells were cultured in hormone‐depleted medium ± E2 for 4 weeks; then, cells were fixed and stained with crystal violet for colony quantification using imagej. (E) MCF‐7 and FR cells seeded at low density were treated ± fulv. After 4 weeks, colonies were quantified as in (D). In (D, E), data are shown as mean of triplicates + SD relative to parental control. *P ≤* *0.05 by *t*‐test.

ER+ breast tumors classically require estrogen‐induced ER signaling for growth; however, clinical studies investigating estrogen therapies have demonstrated that tumors growing in low‐estrogen conditions may be growth‐inhibited by estrogen (Ellis *et al*., 2009). To identify the mechanism underlying the anticancer effects of estrogen in ER+ breast cancer, we first evaluated three preclinical tumor models. WHIM16 patient‐derived xenografts (PDXs) were derived from a patient treated with multiple lines of endocrine therapy and chemotherapy. This patient experienced partial tumor regression in response to E2 treatment, at which point a skin metastasis was harvested for PDX development (Puenpa *et al*., 2013). Ovariectomized NSG mice bearing serially transplanted WHIM16 tumors were treated ± E2. E2 induced rapid and durable tumor regression (Fig. 1A and Fig. S1A,B).

Long‐term treatment of BALB/c mice with medroxyprogesterone acetate (MPA) induces the formation of ER+ mammary adenocarcinomas, which were serially transplanted until progestin‐independent variants (e.g., C7‐2‐HI, C4‐HI) were established (Kordon *et al*., 1991; Soldati *et al*., 2010; Vanzulli *et al*., 2005). C7‐2‐HI and C4‐HI tumors were serially transplanted into ovariectomized BALB/cJ mice. E2 treatment of C7‐2‐HI tumor‐bearing mice induced partial but durable regression (Fig. 1B and Fig. S1C,D). In contrast, C4‐HI tumors were growth‐inhibited by E2 treatment but did not consistently regress (Fig. 1C and Fig. S1E,F).

To provide preclinical models more amenable to genetic manipulation, we also evaluated two types of anti‐estrogen‐resistant *in vitro* systems: (a) MCF‐7 cells with acquired resistance to the selective ER downregulator fulv (FR) were developed by maintenance in medium containing 1 μm fulv for > 1 year; (b) MCF‐7 cells with acquired resistance to long‐term estrogen deprivation (LTED), which mimics the estrogen depletion induced by AI therapy in patients, were developed by maintenance in hormone‐depleted medium for > 1 year. Treatment with 1 nm E2 increased growth of parental MCF‐7 cells but suppressed growth of LTED cells (Fig. 1D). Similarly, fulv treatment suppressed growth of parental MCF‐7 cells, while fulv withdrawal (FW) suppressed growth of FR cells (Fig. 1E).

### 3.2 Estrogen‐induced apoptosis is associated with nuclear ER transcriptional activation

**Figure 2:**
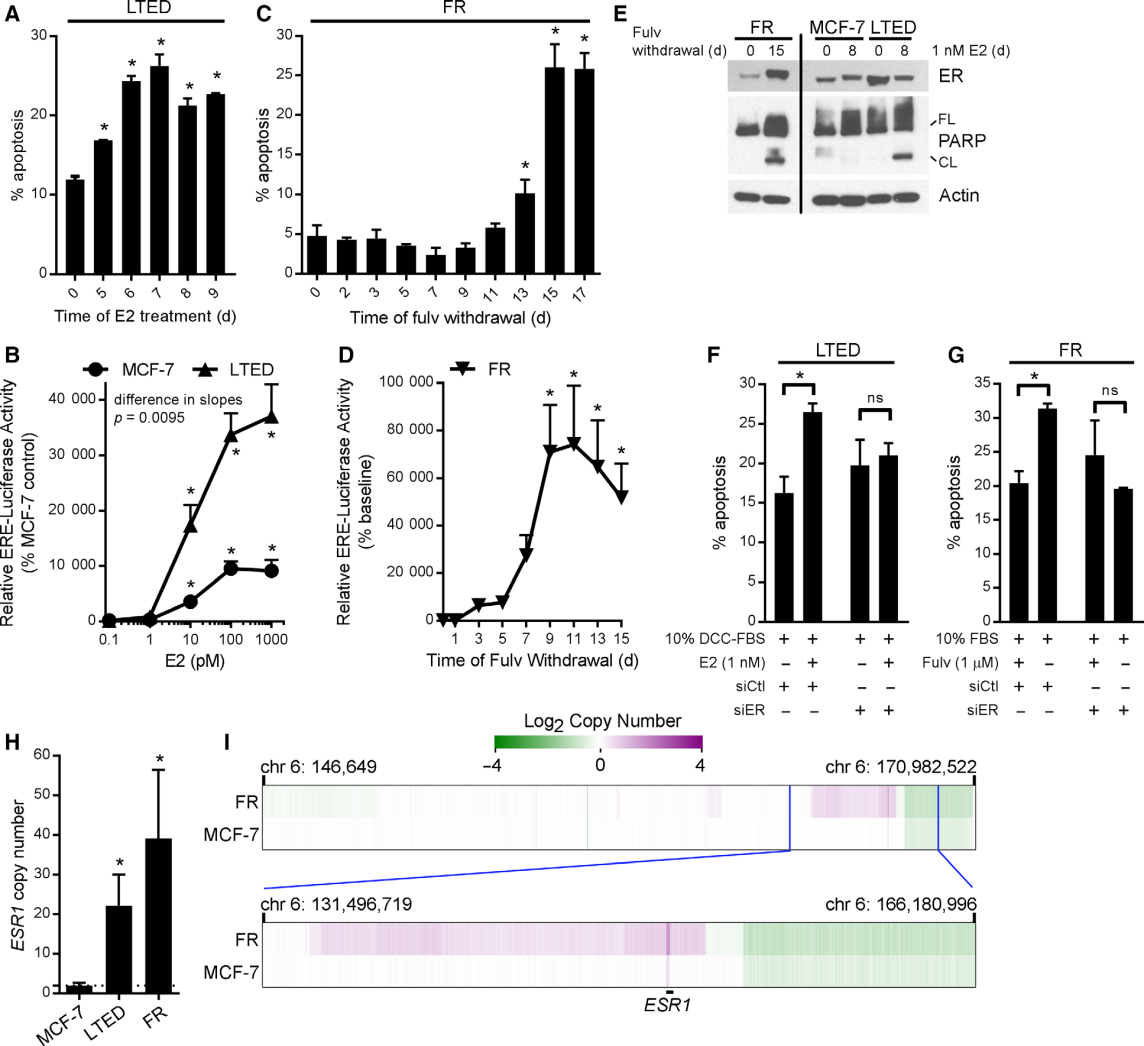
Restoration of ER signaling drives cell death in MCF‐7 cells with acquired resistance to fulvestrant or long‐term estrogen deprivation. (A) LTED cells were cultured in hormone‐depleted medium ± 1 nm E2 for up to 9 days, and then, cells were reseeded. Three days later, apoptosis was assayed using annexin and propidium iodide staining followed by flow cytometry. Mean of triplicates + SD is shown. *P ≤* *0.05 by Bonferroni multiple comparison‐adjusted *post hoc* test compared to hormone‐deprived controls. (B) MCF‐7 and LTED cells were transfected with ERE‐driven firefly luciferase and CMV‐Renilla. The next day, cells were treated ± E2, and luciferase activities were measured 24 h later. Firefly signal was normalized to Renilla signal, and data are shown as mean of triplicates + SD relative to baseline within each cell line. *P ≤* *0.05 by Bonferroni multiple comparison‐adjusted *post hoc* test compared to baseline within each cell line. Curves were compared by linear regression. (C) Fulv was withdrawn from FR cells for up to 15 days, and then, cells were reseeded. Three days later, apoptosis was assayed as in (A). (D) FR cells were treated as in (C), and then, cells were transfected as in (B). Luciferase activities were measured 2 days post‐transfection, and data were analyzed as in (B). (E) Immunoblot analysis of lysates from FR cells treated ± fulv, or MCF‐7 and LTED cells treated ± E2. All lanes were captured from the same blot exposure for each protein. FL, full length; CL, cleaved. (F) LTED cells were transfected with siRNA targeting ER (or nonsilencing control). After 4 days of treatment ± E2, cells were assayed for apoptosis as in (A). *P ≤* *0.05 by *t*‐test. (G) FR cells were treated ± fulv for 10 days, then transfected with siRNA, and assayed as in (F). (H) Primers targeting *ESR1* and *ASXL2* (control) were used for qPCR of genomic DNA. Ratio of *ESR1/ASXL2* (ΔΔ*C*_t_) was normalized to MCF‐7 cells. *P ≤* *0.05 by *t*‐test. (I) DNA from MCF‐7 and FR cells was analyzed using genome‐wide SNP arrays. DNA copy number results from the region on chromosome 6 containing *ESR1* are indicated at low (top) and high (bottom) resolutions.

E2 induced ER transcriptional reporter activity and apoptosis that peaked after 7 days of exposure in LTED cells (Fig. 2A,B). FW from FR cells induced apoptosis after ~ 13 days (Fig. 2C), which was preceded by elevated ER transcriptional activity that increased by Day 7 and peaked near Day 11 following FW (Fig. 2D). Proteins encoded by ER‐inducible genes (e.g., IRS‐1, IGF‐1R) were similarly increased after 6–10 days of FW (Fig. S2). Apoptotic effects of E2 and FW were confirmed by immunoblot analysis of PARP cleavage (Fig. 2E). siRNA knockdown of ER prevented apoptosis induced by FW in FR cells and by E2 in LTED cells (Fig. 2F,G and Fig. S3A,B), confirming that ER is required for apoptotic effects of FW and E2. Compared to parental MCF‐7 controls, fulv‐withdrawn FR cells and hormone‐deprived LTED cells had higher ER protein levels (Fig. 2E and Fig. S2), likely due to genomic amplification of the gene encoding ER, *ESR1* [Fig. 2H,I and ref. (Puenpa *et al*., 2013)]. The WHIM16 PDX model also harbors *ESR1* amplification (Puenpa *et al*., 2013), and *ESR1* amplification was detected in a primary breast tumor from a patient with ER+ metastatic disease that regressed in response to E2‐based therapy (Kota *et al*., 2017). Thus, excessive engagement of ER may promote anticancer effects of estrogens. This concept is further supported by observations from T47D breast cancer cells with acquired resistance to fulv (T47D/FR), which showed suppression of ER expression, and growth was unaffected by FW (data not shown). C7‐2‐HI tumors exhibited only slight amplification of *ESR1* (mean ± SD of 1.39‐fold ± 0.17‐fold; *P *=* *0.036 compared to liver control) while C4‐HI tumors did not, potentially contributing to the difference in sensitivity to E2 (Fig. 1B,C and Fig. S1C–F) and suggesting that *ESR1* amplification may not be required for therapeutic response to E2.

In ER transcriptional reporter activity assays, LTED cells showed hypersensitivity to E2 compared to parental MCF‐7 cells (Fig. 2B), concurrent with prior observations (Miller *et al*., 2011; Song *et al*., 2001). While MCF‐7 and LTED cells were growth‐stimulated by 1 pm E2, higher doses (10 pm to 1 nm) induced and suppressed growth in parental and LTED cells, respectively (Fig. S4). Human physiological serum E2 levels reach > 1 nm in premenopausal women and ~ 40 pm in postmenopausal women, suggesting that physiological E2 levels may be sufficient to inhibit growth of ER+ breast cancer cells with acquired resistance to hormone deprivation (Baird and Fraser, 1974; Iwase *et al*., 2013; Yao *et al*., 2000). The growth‐suppressive effect of E2 in LTED cells was acute: Just 1 h of treatment with 1 nm E2 (followed by a 25‐day estrogen‐free period) was sufficient to stunt growth, and 24 h of E2 treatment elicited maximal long‐lasting growth inhibition in a 4‐week assay (Fig. S5A). Measurement of levels of ER‐inducible transcripts indicated that ER activity returned to baseline within 4 days of E2 withdrawal in MCF‐7 cells (Fig. S5B). Thus, reduction of the duration of treatment may lead to equivalent efficacy while limiting adverse events in patients treated with exogenous estrogens.

Although the best‐characterized role of ER is as a transcription factor, ER has been reported to have nongenomic effects including membrane‐initiated signaling. Other estrogen‐binding proteins have also been implicated in cytoplasmic signaling (Miller *et al*., 2009; Revankar *et al*., 2005; Song *et al*., 2006). Treatment of parental MCF‐7 cells with 1 nm E2 for 4 h decreased cytoplasmic levels of ER but did not appreciably alter nuclear ER; in contrast, E2 increased cytoplasmic ER levels in LTED cells (Fig. S6A). To test whether cytoplasmic ER activation is involved in E2‐induced apoptosis in LTED cells, cells were treated ± 1 nm E2 or an equivalent concentration (20 nm) of an EDC that can enter the cytoplasm of cells but not nuclei (Harrington *et al*., 2006). The EDC did not induce ER transcriptional activity and actually increased growth of LTED cells, confirming that E2‐induced apoptosis requires nuclear ER signaling (Figs S4 and S6B).

### 3.3 Estrogen‐induced apoptosis requires activation of an unfolded protein response

**Figure 3:**
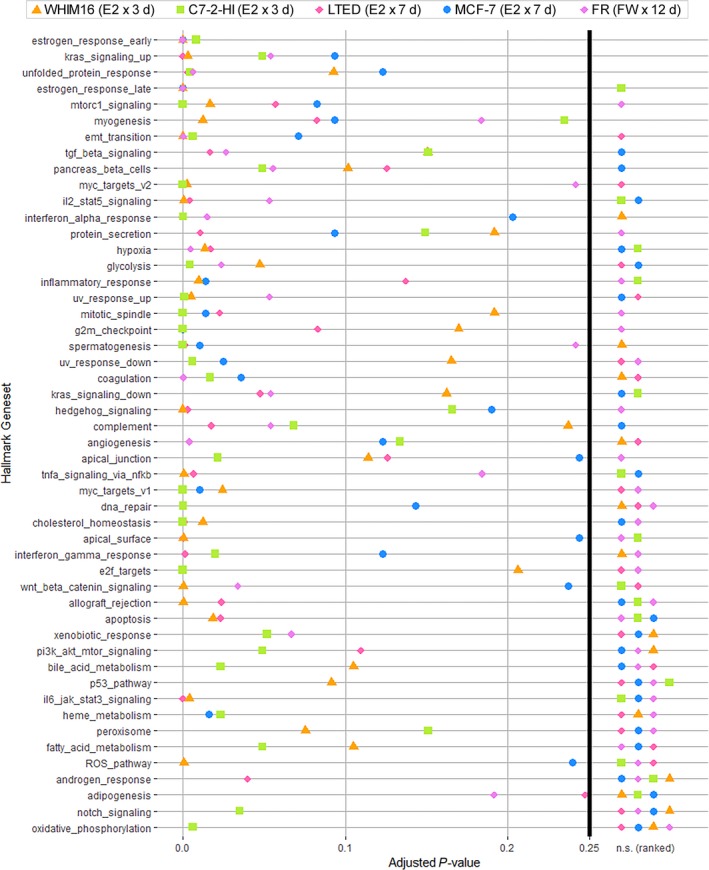
Transcriptomic profiling reveals a UPR upon ER reactivation via E2 treatment or anti‐estrogen withdrawal. RNA was extracted from WHIM16 and C7‐2‐HI tumors from mice treated ± E2 (via s.c. pellet) for 3 days, from MCF‐7 and LTED cells treated ± 1 nm E2 for 7 days, and from FR cells treated ± FW for 12 days, all in triplicate. FR cell RNA was analyzed by gene expression microarray. RNA from other samples was analyzed by sequencing. Whole‐transcriptome expression profiles of E2‐treated and FW‐treated samples compared to baseline were analyzed by unsupervised sample‐wise enrichment analysis using the hallmark geneset collection in GSVA. Adjusted *P*‐values below the significance threshold of 0.25 (Subramanian *et al*., 2005) are shown, and nonsignificant (n.s.) *P*‐values are ranked at right.

**Figure 4:**
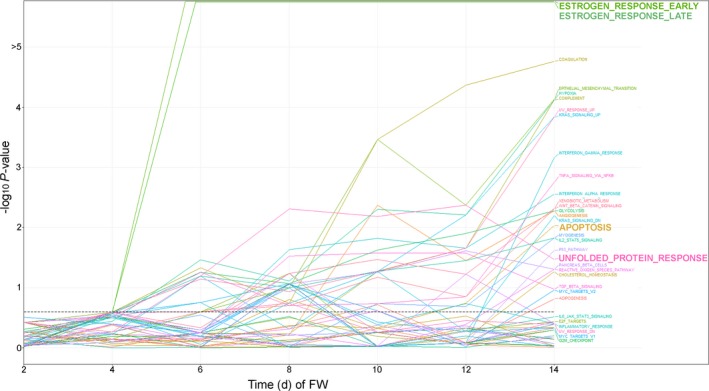
Temporal analysis indicates that ER reactivation precedes a UPR upon anti‐estrogen withdrawal. FR cells were treated ± FW for 0–14 days. RNA was harvested in triplicate in 2‐day intervals for gene expression microarray analysis. Whole‐transcriptome expression profiles of FW‐treated samples compared to baseline were analyzed by unsupervised sample‐wise enrichment analysis of using the hallmark geneset collection in GSVA. Adjusted *P*‐values are shown; the significance threshold of 0.25 (Subramanian *et al*., 2005) is indicated by dotted horizontal line.

Since nuclear ER activity is required for therapeutic effects of ER reactivation (Fig. S4), we performed gene expression profiling to identify genes associated with therapeutic effects of estrogen in preclinical models. After identifying Day 7 as the time point of maximal E2‐induced apoptosis in LTED cells (Fig. 2A), RNA‐seq was performed on MCF‐7 and LTED cells treated ± 1 nm E2 for 7 days. We also performed RNA‐seq of WHIM16 and C7‐2‐HI tumors from mice treated ± E2 (via s.c. pellet) for 3 days (before regression became evident, typically around Day 7). GSVA revealed significant enrichment for genes involved in estrogen response and unfolded protein response (UPR) (Fig. 3). To determine the sequence of activation of these pathways, temporal gene expression profiling was performed using microarrays for FR cells treated with FW for 0, 2, 4, 6, 8, 10, 12, or 14 days. GSVA of FR cell gene expression profiles at individual time points following FW revealed sequential activation of estrogen response at Day 4 (*P *=* *1.4 × 10^−7^ and 8.7 × 10^−7^ for early and late estrogen response gene sets, respectively), followed by a UPR at Day 8 (*P *=* *0.005), and apoptosis at Day 14 (*P *=* *0.009) (Figs 3 and 4). Together, these data suggest that the mechanism of ER reactivation‐induced apoptosis involves activation of a UPR.

**Figure 5:**
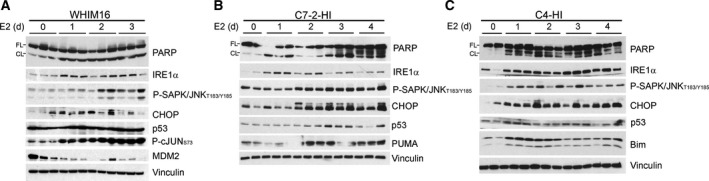
Estrogen‐independent tumors exhibit a UPR in response to E2 treatment. Mice bearing WHIM16 (A), C7‐2‐HI (B), or C4‐HI (C) tumors were treated with E2 via s.c. pellet. Lysates from tumors harvested after 0–4 days were analyzed by immunoblot. FL, full length; CL, cleaved.

**Figure 6:**
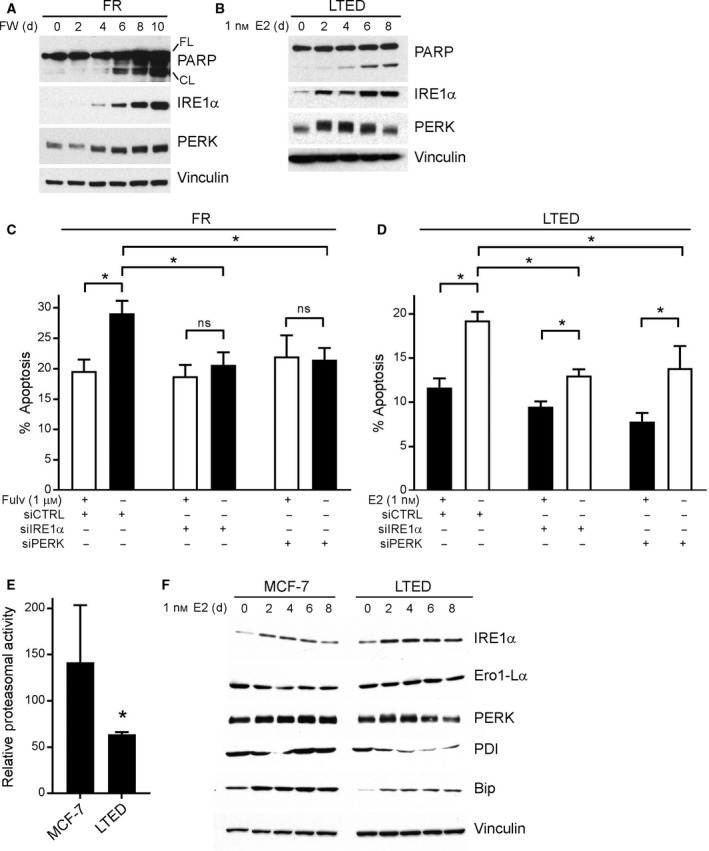
A UPR is required for apoptotic response to ER reactivation. (A, B) Lysates from FR cells treated with FW for 0–10 days (A) and from LTED cells treated with E2 for 0–8 days (B) were analyzed by immunoblot. FL, full length; CL, cleaved. (C) FR cells were treated ± FW for 10 days, then reseeded, and transfected with siRNA targeting IRE1α, PERK, or nonsilencing control. Four days later, cells were assayed for apoptosis. Mean of triplicates + SD is shown. *P ≤* *0.05 by Bonferroni multiple comparison‐adjusted *post hoc* test. (D) LTED cells were transfected with siRNA as in (C) and then treated ± E2 for 4 days prior to assay as in (C). (E) MCF‐7 and LTED cells were treated with hormone‐depleted medium for 4 days and then assayed for proteasomal activity using the Promega Proteasome‐Glo Luciferase Kit. Mean of triplicates + SD is shown. *P ≤* *0.05 by *t*‐test. (F) MCF‐7 and LTED cells were treated with E2 for 0–8 days, and then, lysates were analyzed by immunoblot.

E2 treatment induced upregulation of the unfolded protein sensor IRE1α and the pro‐apoptotic proteins CHOP, PUMA, and/or Bim in WHIM16, C7‐2‐HI, and C4‐HI tumors (Fig. 5). Similarly, levels of IRE1α and the unfolded protein sensor PERK were increased following FW and E2 treatment in FR and LTED cells, respectively, which preceded apoptosis (indicated by PARP cleavage; Fig. 6A,B); such changes were paralleled by increases in Bim and CHOP (Fig. S7). The protein‐folding chaperone Bip (GRP78) has been shown to bind and inhibit cleavage of procaspase 7, inhibit activity of the pro‐apoptotic BCL‐2 family protein Bik, and increase levels of anti‐apoptotic BCL2 family proteins, which converge to suppress apoptosis and promote resistance to anticancer drugs including anti‐estrogens (Cook *et al*., 2012; Fu *et al*., 2007; Reddy *et al*., 2003). EnR stress can also induce increases in levels of the pro‐apoptotic BCL‐2 family proteins Bim, PUMA, and NOXA (Puthalakath *et al*., 2007; Wang *et al*., 2016).

To establish a requirement for the UPR for apoptosis in response to ER reactivation, siRNA knockdown of the primary apoptotic UPR mediators IRE1α and PERK was performed in conjunction with FW or E2 treatment in FR and LTED cells, respectively. Knockdown of IRE1α or PERK provided complete protection against FW‐induced apoptosis in FR cells, and partial protection against E2‐induced apoptosis in LTED cells, confirming that activation of a prolonged UPR by ER reactivation contributes to cell death (Fig. 6C,D and Fig. S3C–F).

To further investigate the mechanism underlying ER reactivation‐induced UPR and apoptosis, the relative levels of UPR mediators, protein‐folding chaperones, and proteasomal activity between parental MCF‐7 and LTED cells were measured. Compared to hormone‐deprived MCF‐7 cells, LTED cells had less basal proteasomal activity (Fig. 6E); this difference may contribute to the protein‐folding stress in LTED cells upon E2 stimulation. In addition, LTED cells expressed lower levels and showed weaker ability to induce expression of the chaperones Bip and PDI in response to E2 compared to MCF‐7 cells (Fig. 6F).

### 3.4 p53 and JNK signaling are required for apoptosis induced by ER reactivation

**Figure 7:**
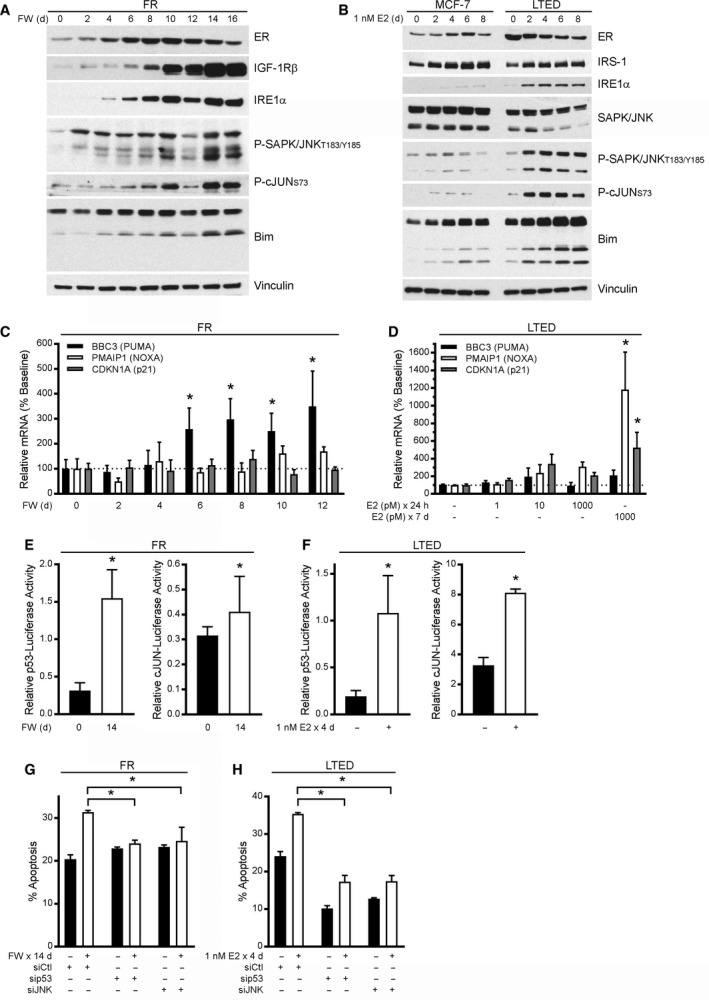
ER reactivation‐induced apoptosis is dependent upon activation of p53 and JNK pathways. (A, B) FR cells were treated with FW for 0–16 days (A), and MCF‐7 and LTED cells were treated with E2 for 0–8 days (B). Lysates were analyzed by immunoblot. (C, D) RNA was harvested from FR cells following 0–12 days of FW (C) or from LTED cells following treatment with E2 (D). RNA was analyzed by RT‐qPCR, and signal values for the indicated transcripts were normalized to 36B4 housekeeping transcript values (ΔΔ*C*_t_). (E) FR cells were treated ± FW for 10 days and then transfected with p53‐ or cJUN‐driven firefly luciferase and CMV‐Renilla (control). Firefly activity was measured 4 days after transfection and normalized to Renilla activity. (F) LTED cells were transfected as in (E) and then treated ± E2 for 4 days before measuring luciferase activities as in (E). (G) FR cells were treated ± FW for 10 days and then transfected with siRNA. Apoptosis was measured 4 days later. (H) LTED cells were transfected as in (G), treated ± E2 for 4 days, and analyzed as in (G). In (C–H), data are shown as mean of triplicates + SD. In (C, D, G, H), *P ≤* *0.05 by Bonferroni multiple comparison‐adjusted *post hoc* test compared to baseline unless otherwise indicated. In (E, F), *P ≤* *0.05 by *t*‐test.

The anticancer effects of activating the UPR have been linked to induction of multiple other pathways, including the p53, stress‐activated protein kinase/Jun amino‐terminal kinase (SAPK/JNK), mechanistic target of rapamycin complex I (mTORC1), and nuclear factor κ‐light‐chain‐enhancer of activated B cells (NFκB) pathways (Hotamisligil, 2010). We found that therapeutic effects of E2 were associated with increased activation of the JNK pathway (assessed by increased P‐JNK and P‐cJUN) and the p53 pathway (assessed by increased p53 and decreased MDM2) upon E2 treatment in tumor models and LTED cells, and upon FW in FR cells (Figs 5 and 7A,B; MDM2 was not detected in murine tumors). FW and E2 treatment drove upregulation of p53‐driven transcripts encoding PUMA, NOXA, and p21 (Fig. 7C,D) in FR and/or LTED cells, respectively, at time points consistent with induction of apoptosis (Fig. 2A,C). Transcriptional reporter assays confirmed activation of both p53 and cJUN in FR and LTED cells following ER reactivation (Fig. 7E,F). Furthermore, activation of cJUN was dependent upon the UPR sensor IRE1α, as treatment with the IRE1α inhibitor KIRA6 blocked E2‐induced cJUN transcriptional activity in LTED cells (Fig. S7D). Importantly, p53 and JNK were required for FW‐ and E2‐induced apoptosis, as siRNA knockdown of p53 or JNK prevented apoptosis (Fig. 7G,H and Fig. S3G,H).

NFκB and mTORC1 signaling were not found to be activated upon E2 treatment (in LTED cells) or FW (in FR cells). Instead, immunoblotting and transcriptional reporter assays revealed downregulation of NFκB‐inducible transcripts and encoded proteins in response to ER reactivation. Markers of mTORC1 activity (P‐S6 and P‐p70S6K) were either downregulated or unchanged upon ER reactivation (Fig. S8).

## 4 Discussion

Estrogen is classically considered a tumor promoter in ER+ breast cancer; thus, there is hesitation to implement E2 as a common therapeutic option, even in tumors entirely resistant to anti‐estrogens. However, we and others have demonstrated that long‐term adaptation to estrogen deprivation or anti‐estrogens can sensitize ER+ breast cancer cells and tumors to the cytotoxic effects of ER reactivation. The successful resurrection of estrogen therapy in the clinic would be enhanced by (a) understanding of the underlying molecular mechanism, which would (b) facilitate the development of a biomarker to identify patients likely to benefit. Herein, we demonstrated that restoration of ER signaling drives cancer cell death mediated by the UPR, offering protein‐folding stress as an avenue to identify a potential biomarker and a potential complementary therapeutic opportunity.

Long‐term estrogen‐deprived and FR cells, as well as WHIM16 patient‐derived xenografts and C7‐2‐HI murine mammary adenocarcinoma allografts that regress upon E2 treatment, exhibit genomic amplification of *ESR1*. In contrast, *ESR1* is not amplified in C4‐HI tumors, which are only partially responsive to E2 therapy [Figs 1 and 2H,I, Fig. S1, and ref. (Puenpa *et al*., 2013)]. Thus, hypersensitivity to E2 and subsequent ER‐mediated cell death may be enhanced in tumors harboring genomic amplification of *ESR1*. *ESR1* amplification has been observed in up to 20% of metastatic ER+ breast tumors from patients (Holst, 2016; Lefebvre *et al*., 2016; Lin *et al*., 2013). Importantly, a case study showed that E2 therapy caused partial tumor regression in a patient with *ESR1*‐amplified metastatic breast cancer (Kota *et al*., 2017). *ESR1* amplification was also detected in the metastatic skin lesion of the patient from whom the WHIM16 PDX model was created, and this patient experienced a ‘modest response’ to E2 therapy (Puenpa *et al*., 2013). These observations suggest that *ESR1* amplification may be a tumor biomarker to enrich for the subpopulation of patients likely to benefit from E2 therapy.

In three E2‐sensitive tumor models, and in LTED and FR cells, ER reactivation induced apoptosis and tumor regression concomitant with a UPR and activation of p53 and JNK. Prior studies showed that the hormone‐independent MCF‐7:5C subline expresses higher levels of JNK and P‐JNK than parental MCF‐7 controls and that P‐JNK levels are further induced by E2 treatment in 5C cells. Although treatment with the JNK inhibitor SP600125 does not block E2‐induced apoptosis in 5C cells (Fan *et al*., 2015), a supraphysiologic dose of E2 (20 μm) causes apoptosis of parental MCF‐7 cells that is blocked by cotreatment with SP600125 (Altiok *et al*., 2007). In contrast, we observed that JNK levels and activation are similar in hormone‐deprived MCF‐7 and LTED cells, but both markers were robustly induced by E2 in LTED cells and tumor models, and by FW in FR cells (Figs 5 and 7A/B). siRNA‐induced knockdown of JNK protected LTED and FR cells against ER reactivation‐induced death (Fig. 7G,H), suggesting that JNK activation contributes to apoptosis. Thus, basal JNK expression may not be a good biomarker to predict response to estrogen therapy, but activation of JNK may be useful as an early pharmacodynamic biomarker of response.

Prior studies suggested an association between therapeutic effects of E2 and EnR stress in ER+ breast cancer cells *in vitro*. Gene expression microarray analysis of cells that are growth‐stimulated (MCF‐7) vs. growth‐inhibited (MCF‐7:5C) by E2 revealed E2‐induced upregulation of EnR stress genes and inflammatory response genes selectively in 5C cells (Ariazi *et al*., 2011; Fan *et al*., 2014). E2 also induced expression of pro‐apoptotic proteins that contributed to E2‐mediated apoptosis, including Bax and Bim; siRNA knockdown of Bax or Bim, and to a lesser degree p53, attenuated E2‐induced apoptosis in 5C cells (Lewis *et al*., 2005). Early studies with C7‐2‐HI tumors also demonstrated that E2‐induced regression was associated with expression of the p53 target genes encoding p21 (*Cdkn1a*) and p27 (*Cdkn1b*) (Vanzulli *et al*., 2002, 2005). Our studies confirmed that p53 is a critical mediator of E2‐induced death in FR and LTED cells, and that this pathway is activated by E2 in WHIM16, C4‐HI, and C7‐2‐HI tumors. Thus, loss‐of‐function genomic alterations in p53 (*TP53*), which are observed in approximately 17–20% and 27% of ER+ primary and metastatic breast tumors, respectively (Bertheau *et al*., 2013; Lefebvre *et al*., 2016), may ultimately prove to be contraindicating for use of E2 therapy.

Activation of a UPR in response to E2 in LTED cells was correlated with lower basal proteasomal activity and levels of protein‐folding chaperones (e.g., Bip/GRP78, PDI) (Fig. 6E,F). Bip is a crucial stress sensor of unfolded protein levels in the EnR that binds to and inactivates IRE1α, ATF6, and PERK (Hotamisligil, 2010). When misfolded proteins accumulate, Bip is released from IRE1α, ATF6, and PERK to assist in protein folding, allowing for downstream activation of a UPR by IRE1α/ATF6/PERK. Prior studies established that Bip desensitizes IRE1α to low levels of stress to tailor a UPR based upon stress levels and duration (Pincus *et al*., 2010). Therefore, the Bip deficiency in LTED cells (Fig. 6F) may contribute to IRE1α hyperactivation and consequent apoptosis. ‘Priming’ a UPR that would drive tumor cells toward death (rather than proliferation) during E2 treatment may thus represent a potential therapeutic strategy; for example, the addition of a proteasome inhibitor to E2 treatment may stimulate an enhanced UPR and increase therapeutic efficacy.

## 5 Conclusions

In summary, restoration of ER activity elicited anticancer effects in cell and tumor models of both anti‐estrogen resistance and long‐term estrogen deprivation. The mechanism underlying response to E2 treatment or anti‐estrogen withdrawal is facilitated by IRE1α‐mediated activation of a UPR. This then drives downstream p53 and JNK signaling, which may be used as early indicators of antitumor effects, and subsequent apoptosis. These data also suggest that re‐introduction of E2 to routine clinical use for breast cancer therapy will be most likely to benefit patients with *ESR1* amplification and deficiency in protein‐folding chaperones.

## Conflict of interest

The authors declare no conflict of interest.

## Author contributions

SRH, JDW, NAT, JLF, RAH, ANK, ED, and TWM performed experiments and data analysis. SRH, KS, and TWM interpreted data and wrote the manuscript. All authors read and approved the final manuscript.

## Supporting information


**Fig. S1.** Estrogen‐independent tumors exhibit therapeutic sensitivity to E2 treatment.
**Fig. S2.** MCF‐7/FR cells recover ER signaling following fulv withdrawal.
**Fig. S3.** Confirmation of siRNA knockdown in FR and LTED cells.
**Fig. S4.** LTED cells are hypersensitive to E2 treatment.
**Fig. S5.** Growth‐suppressive effect of E2 on LTED cells is acute.
**Fig. S6.** Anti‐cancer effect of E2 treatment is dependent on nuclear ERα activation.
**Fig. S7.** ER reactivation induces nuclear localization of phospho‐cJUN and activation of pro‐apoptotic CHOP.
**Fig. S8.** NFκB and mTORC1 signaling are not significantly altered following ER reactivation in FR or LTED cells.

## Data Availability

Gene expression microarray data are available at NCBI GEO (accession # GSE121379). SNP array data are available at NCBI GEO (accession # GSE121631). RNA‐seq data are available at NCBI SRA (accession #PRJNA497539).
