# Supplementary material for: Estrogen therapy induces an unfolded protein response to drive cell death in ER+ breast cancer
Source: Mol Oncol. 2019 Jul 9;13(8):1778–94. doi: 10.1002/1878-0261.12528 (PMC6670014; doi:10.1002/1878-0261.12528)

Supplemental Data for Hosford *et al.*

Supplemental Figure 1 – Estrogen-independent tumors exhibit therapeutic sensitivity to E2 treatment. Ovariectomized NSG (A/B) or Balb/c (B-F) mice were implanted s.c. with ~8-mm^3 fragments of serially transplanted WHIM16 patient-derived xenografts (A/B), C7-2-HI murine allografts (C/D), or C4-HI murine allografts (E/F). When tumors reached ~400 mm^3, mice were randomized to sham surgery or s.c. implantation with a 17β-estradiol (E2) pellet. Tumor volume is presented as % change from baseline at the day of E2 supplementation (Day 0). Each line represents one tumor. Summarized data are shown in Fig. 1A-C.

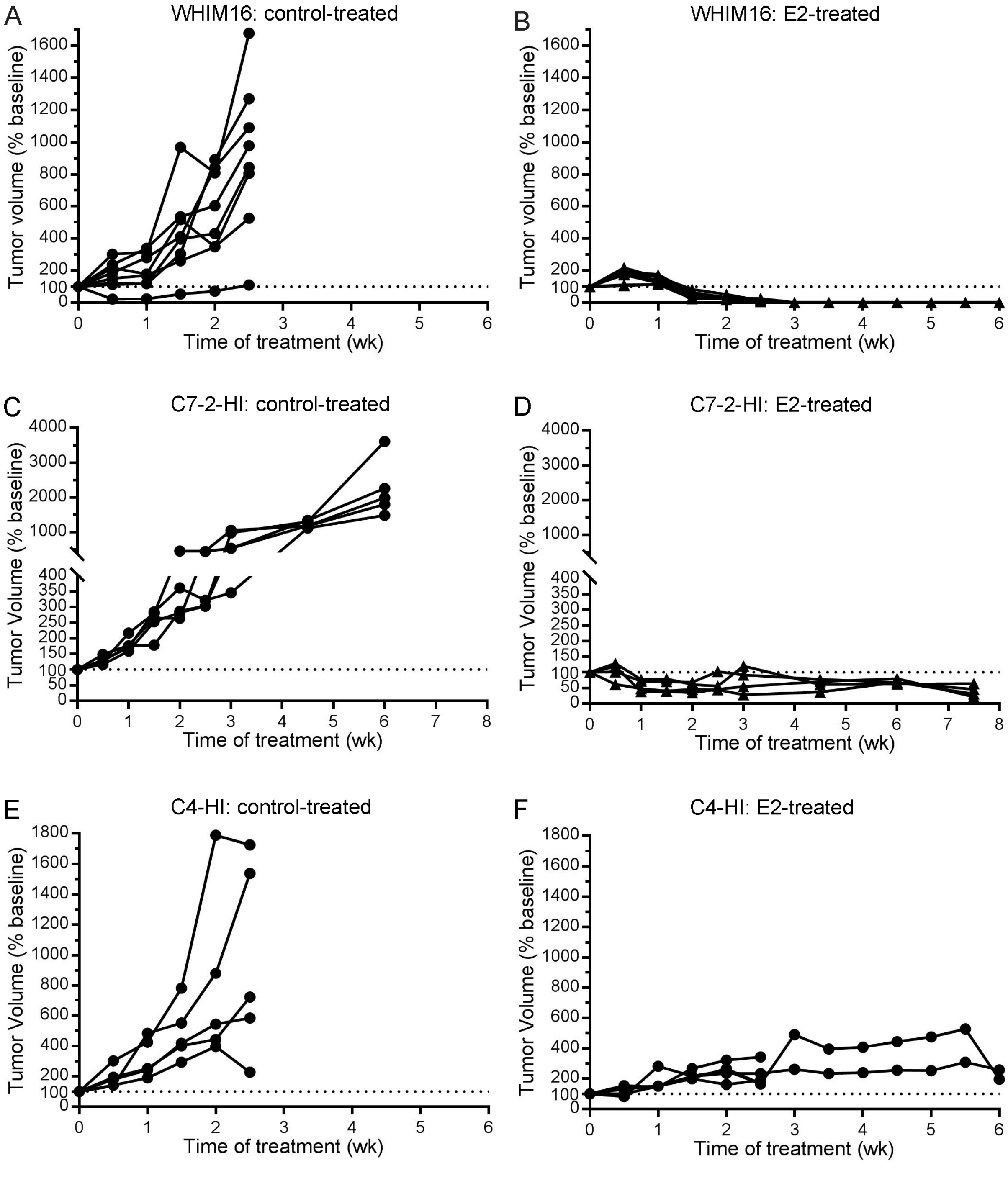

Supplemental Figure 2 - MCF-7/FR cells recover ER signaling following fulv withdrawal. Lysates from MCF-7 and FR cells treated ± fulv for 0-16 d were analyzed by immunoblot.

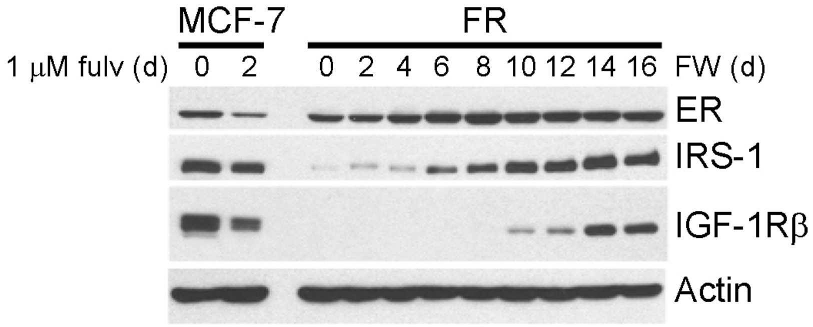

Supplemental Figure 3 – Confirmation of siRNA knockdown in FR and LTED cells. Cells were transfected with siRNA as indicated, and lysates harvested 4 d later were analyzed by immunoblot.

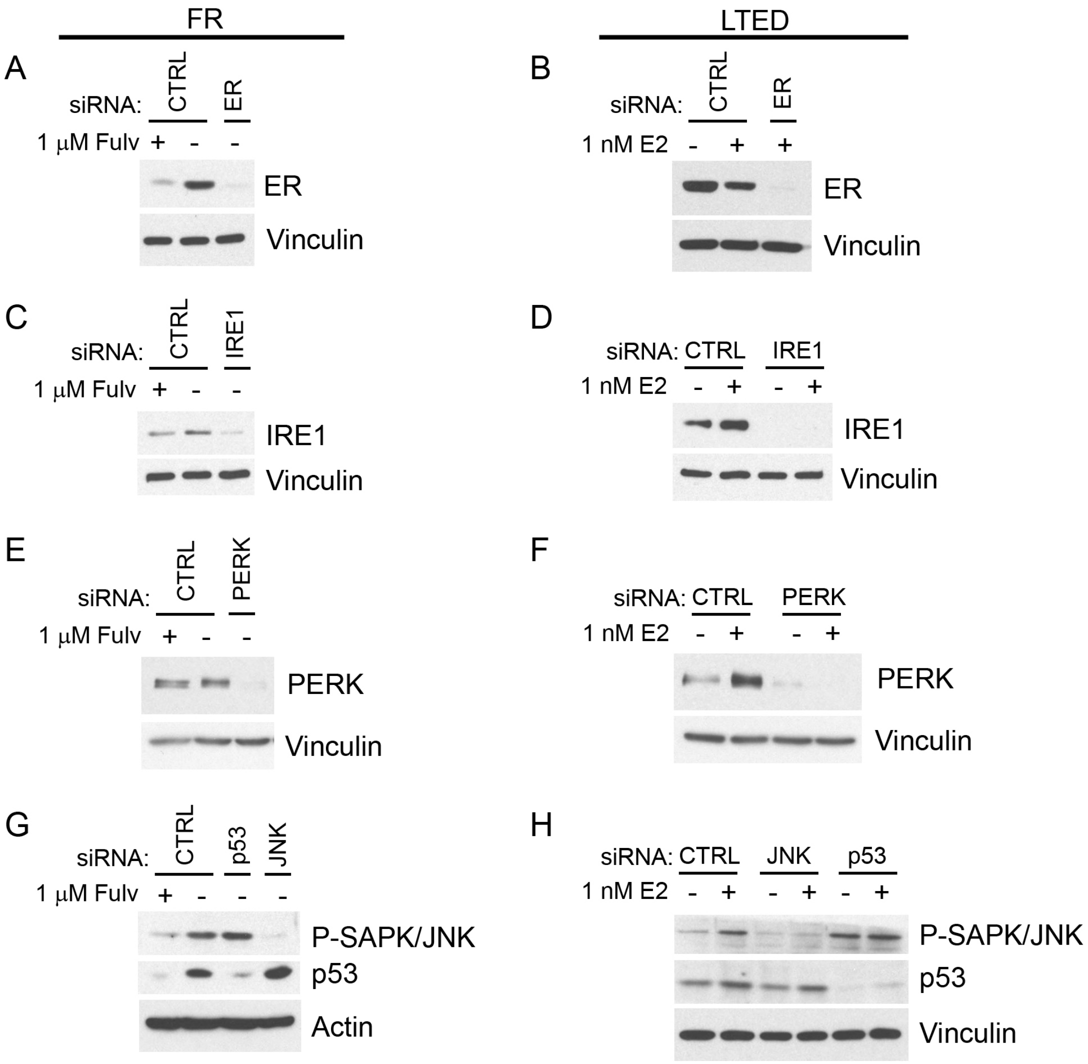

Supplemental Figure 4 - LTED cells are hypersensitive to E2 treatment. MCF-7 and LTED cells were treated ± E2, estrogen-dendrimer conjugate (EDC), or empty dendrimer as indicated for 3 wk, then cells were fixed and stained with crystal violet for colony quantification using the ColonyArea plugin for ImageJ. Data are shown as mean of triplicates ± SD. *$p \leq 0.05$ by Bonferroni multiple comparison-adjusted posthoc test compared to untreated (hormone-deprived) control within each cell line. Representative images are shown.

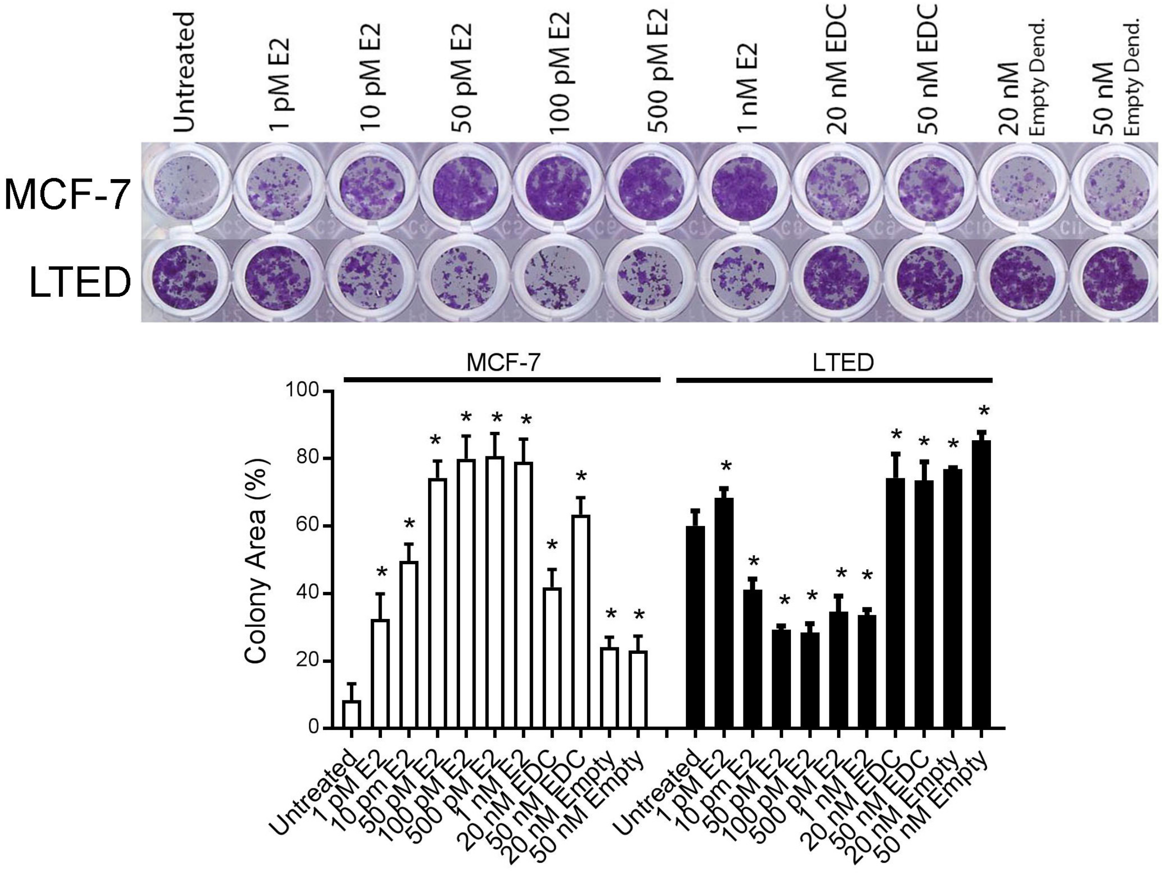

Supplemental Figure 5 – Growth-suppressive effect of E2 on LTED cells is acute. (A) LTED cells were seeded at low density and treated ± E2. Following the indicated time period, E2 was washed out, and cells were allowed to grow in hormone-depleted medium for 4 wk (*top*) or 25 d (*bottom*). Cells were then fixed and stained with crystal violet for colony quantification using the ColonyArea plugin for ImageJ. Data are shown as mean of triplicates ± SD. **p*≤0.05 by Bonferroni multiple comparison-adjusted posthoc test compared to untreated (Day 0) control. (B) To estimate the length of time require for E2-induced ER transcriptional activity to subside following E2 withdrawal, MCF-7 cells were hormone-deprived for 4 d, then treated with ± E2 for 4 d, or for 1 d followed by washout and 1, 2, 3, or 4 d of hormone depletion. On Day 5, RNA was harvested. RT-qPCR was performed using primers targeting *GREB1*, *XBP1,* and *36B4* (control). Ratio of *GREB1/36B4* and *XBP1/36B4* (ΔΔCt) was normalized to baseline. LT- long-term hormone-deprivation for duration of study. **p*≤0.05 by Bonferroni multiple comparison-adjusted posthoc test compared to LT control.

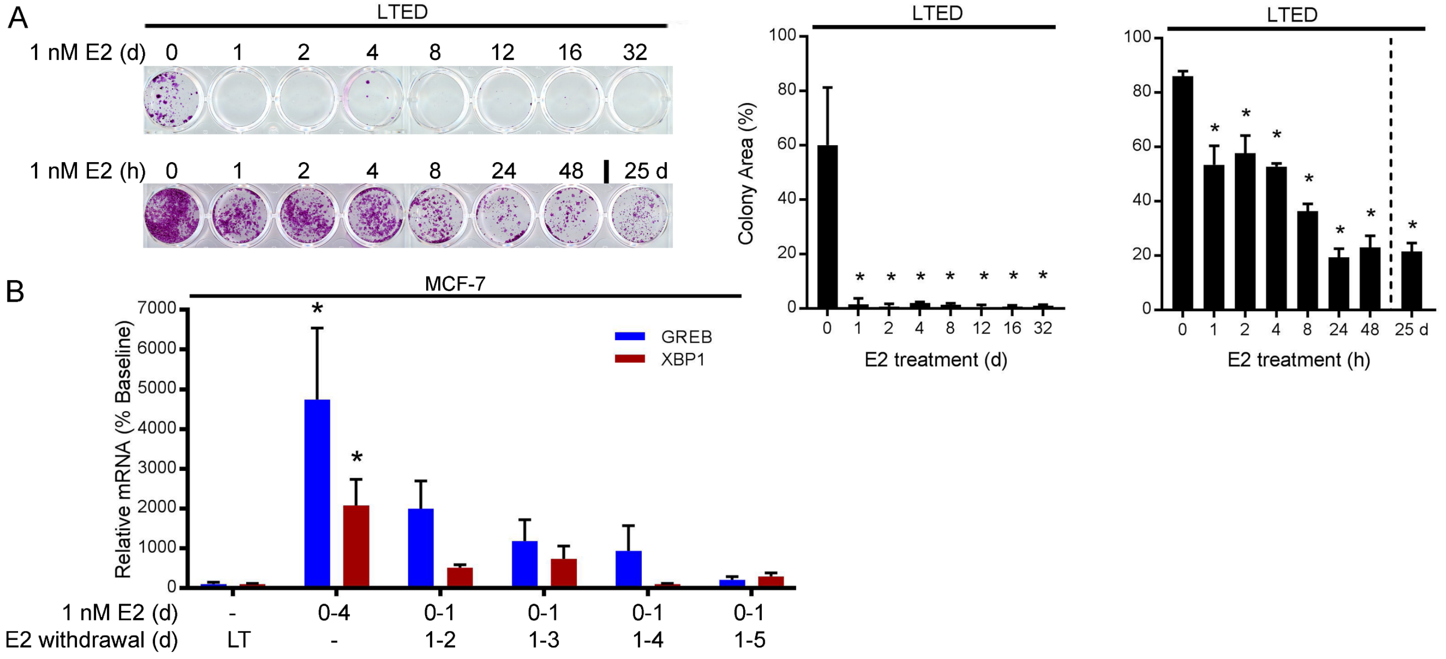

Supplemental Figure 6 - Anti-cancer effect of E2 treatment is dependent on nuclear ERα activation. A) MCF-7 and LTED cells were hormone-starved for 5 d, then treated ± E2 for 4 h. Cells were lysed, and cytoplasmic and nuclear fractions were harvested for analysis by immunoblot. Vinculin and lamin A served as purity/loading controls for cytoplasmic and nuclear fractions, respectively. (B) LTED cells were transfected with plasmids encoding estrogen response element (ERE)-driven firefly luciferase activity and CMV-Renilla. The next day, cells were treated ± E2, estrogen-dendrimer conjugate (EDC), or empty dendrimer. Luciferase activities were measured 2 d later, and firefly was normalized to Renilla. Data are shown as mean of triplicates ± SD. * $p \leq 0.05$ by Bonferroni multiple comparison-adjusted posthoc test compared to untreated control.

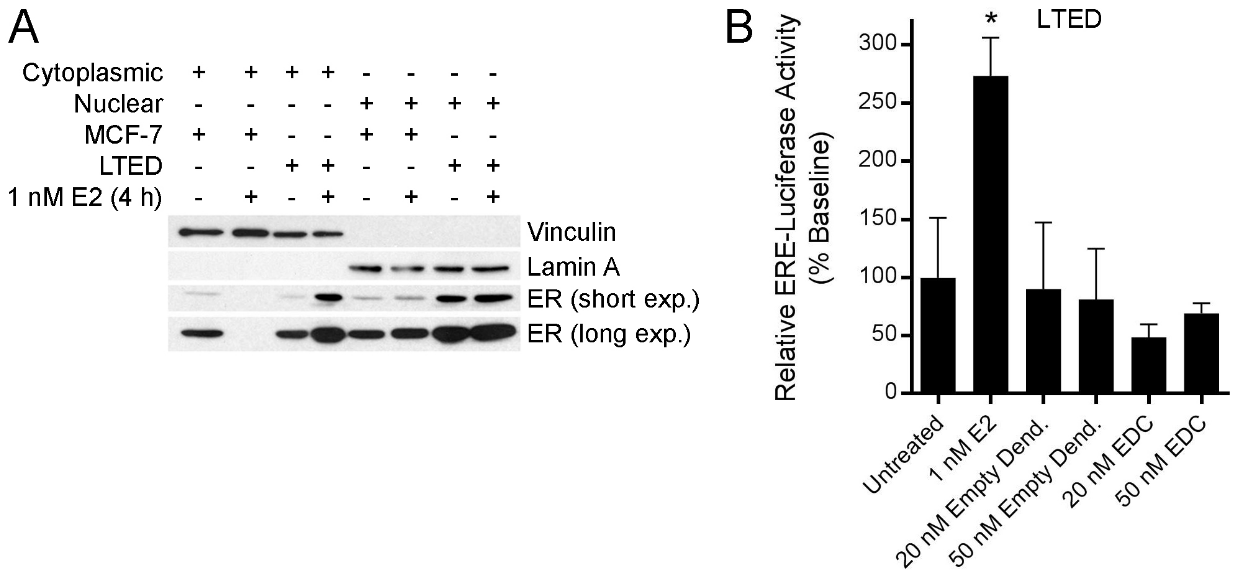

Supplemental Figure 7 – ER reactivation induces nuclear localization of phospho-cJUN and activation of pro-apoptotic CHOP. (A) Immunoblot analysis of lysates from FR cells treated ± FW, or MCF-7 and LTED cells treated ± E2. FL- full length; CL- cleaved. Summarized data are shown in Fig. 2E. FL- full-length; CL-cleaved. (B/C) Immunoblot analysis of cytoplasmic and nuclear fractions harvested from FR cells treated ± FW for 4 d (B), or MCF-7 and LTED cells treated ± E2 for 4 or 24 h (C). Vinculin and lamin A served as purity/loading controls for cytoplasmic and nuclear fractions, respectively. (D) LTED cells were transfected with cJUN-driven firefly luciferase and CMV-Renilla plasmids. The next day, cells were treated ± E2 and the IRE1α inhibitor KIRA6. Luciferase activities were measured 4 d later. Firefly was normalized to Renilla. *$p \leq 0.05$ by Bonferroni multiple comparison-adjusted posthoc test compared to control.

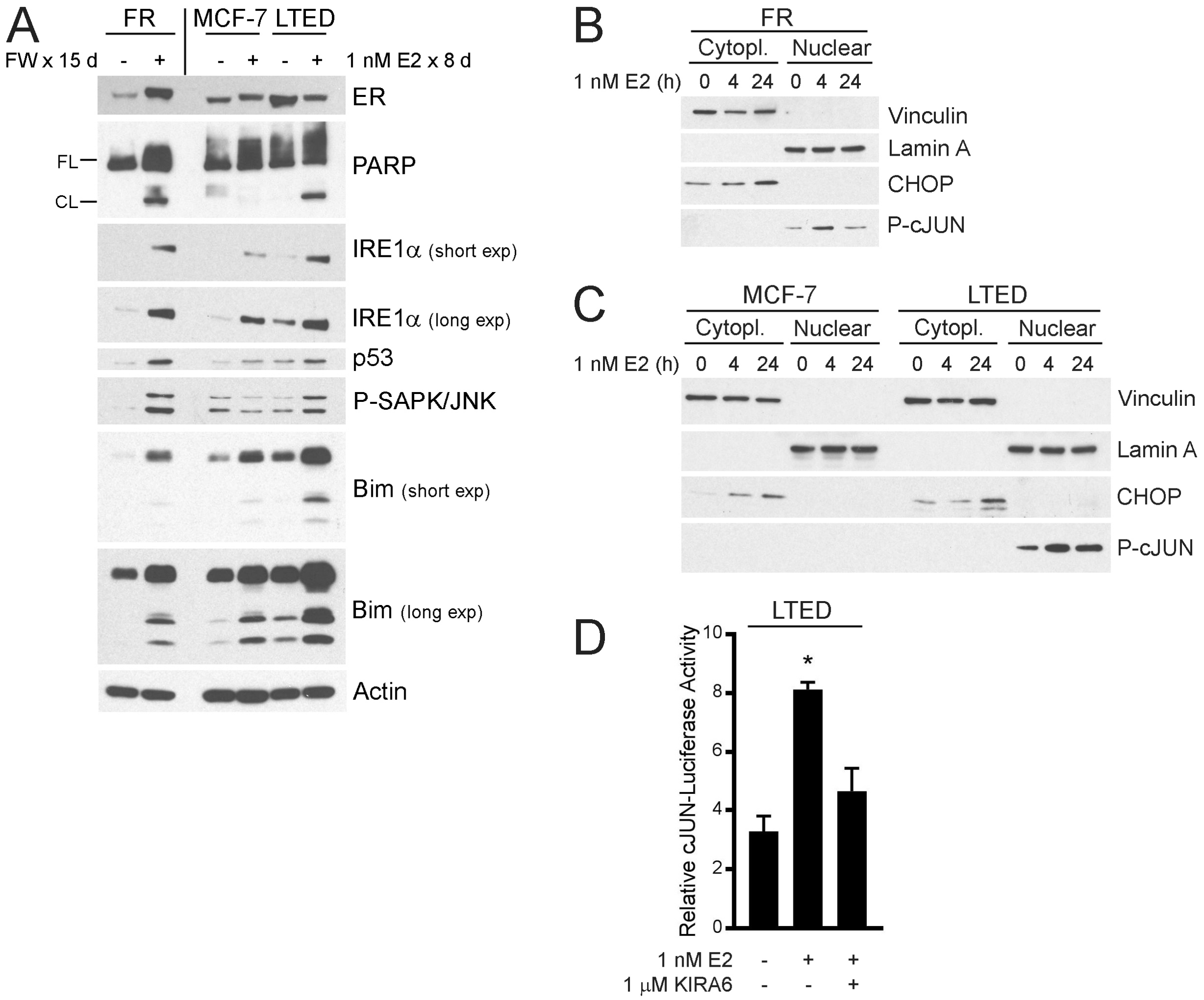

Supplemental Figure 8 - NFκB and mTORC1 signaling are not significantly altered following ER reactivation in FR or LTED cells. (A) MCF-7 and LTED cells were treated with hormone-depleted medium ± E2 for 0-8 d. Lysates were analyzed by immunoblot. (B) Cells were transfected with plasmids encoding NFκB-driven firefly luciferase and CMV-Renilla. Cells were then treated with hormone-depleted medium ± E2. Luciferase activities were measured 3 d later, and firefly was normalized to Renilla. Data are shown as mean of triplicates + SD. Groups were compared by *t*-test. (C) FR cells were treated with FW for 0-16 d. Lysates were analyzed by immunoblot. The same lysates were analyzed in Supplemental Figure 2; actin blot is reproduced here.

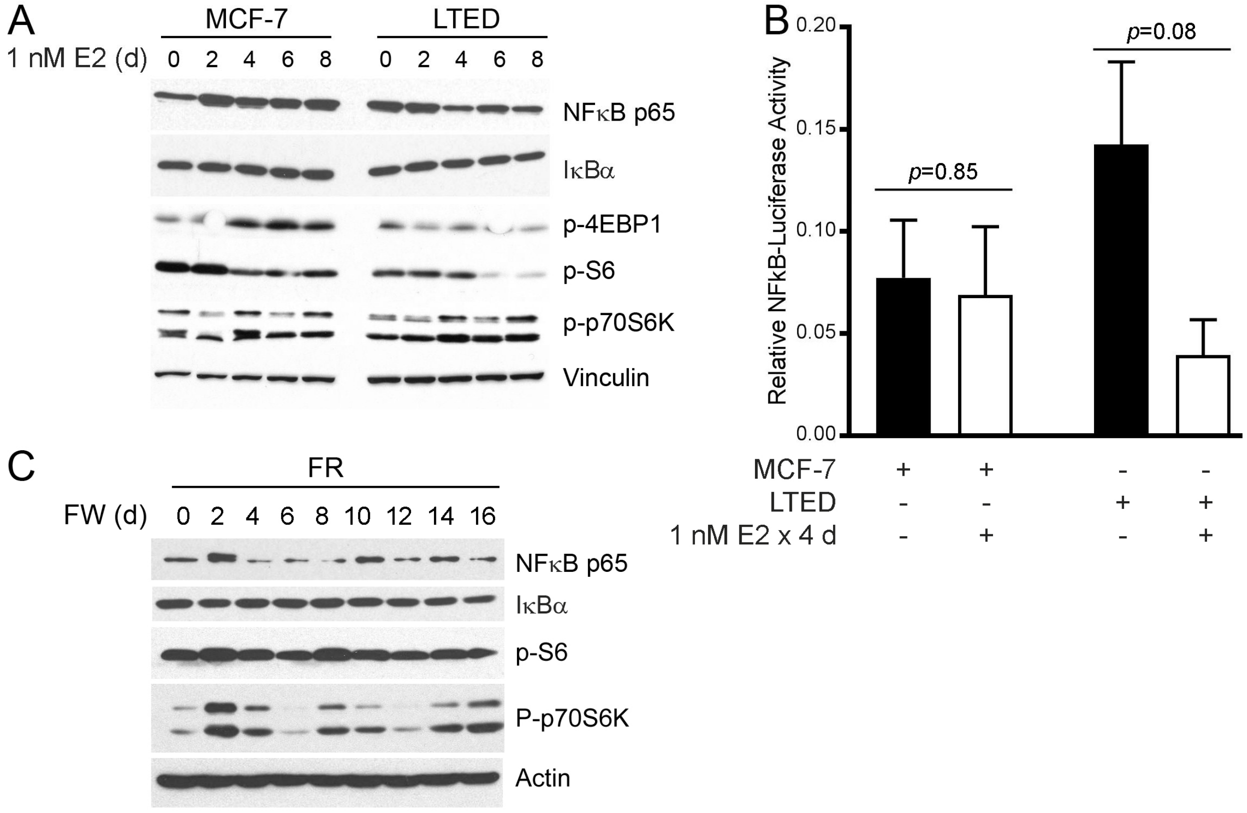

Supplement: Supplementary file 1 — Fig. S1. Estrogen‐independent tumors exhibit therapeutic sensitivity to E2 treatment. Fig. S2. MCF‐7/FR cells recover ER signaling following fulv withdrawal. Fig. S3. Confirmation of siRNA knockdown in FR and LTED cells. Fig. S4. LTED cells are hypersensitive to E2 treatment. Fig. S5. Growth‐suppressive effect of E2 on LTED cells is acute. Fig. S6. Anti‐cancer effect of E2 treatment is dependent on nuclear ERα activation. Fig. S7. ER reactivation induces nuclear localization of phospho‐cJUN and activation of pro‐apoptotic CHOP. Fig. S8. NFκB and mTORC1 signaling are not significantly altered following ER reactivation in FR or LTED cells. [file MOL2-13-1778-s001.pdf]
